# Inhibition of macrophages inflammasome activation *via* autophagic degradation of HMGB1 by EGCG ameliorates HBV-induced liver injury and fibrosis

**DOI:** 10.3389/fimmu.2023.1147379

**Published:** 2023-04-14

**Authors:** Minjing He, Tianhao Chu, Ziteng Wang, Ying Feng, Runhan Shi, Muyang He, Siheng Feng, Lin Lu, Chen Cai, Fang Fang, Xuemin Zhang, Yi Liu, Bo Gao

**Affiliations:** ^1^ Department of Immunology, School of Basic Medical Sciences, Shanghai Medical College of Fudan University, Shanghai, China; ^2^ Department of Dermatology, Shanghai Eighth People’s Hospital, Shanghai, China; ^3^ Department of Trauma Emergency & Critical Care Medicine, Shanghai Fifth People’s Hospital, Fudan University, Shanghai, China; ^4^ Department of Digestive Diseases, Huashan Hospital, Fudan University, Shanghai, China

**Keywords:** liver fibrosis, HBV, EGCG, NLRP3 inflammasome, HMGB1

## Abstract

**Background:**

Liver fibrosis is a reversible wound-healing response that can lead to end-stage liver diseases without effective treatment, in which HBV infection is a major cause. However, the underlying mechanisms for the development of HBV-induced fibrosis remains elusive, and efficacious therapies for this disease are still lacking. In present investigation, we investigated the effect and mechanism of green tea polyphenol epigallocatechin-3-gallate (EGCG) on HBV-induced liver injury and fibrosis.

**Methods:**

The effect of EGCG on liver fibrosis was examined in a recombinant cccDNA (rcccDNA) chronic HBV mouse model by immunohistochemical staining, Sirius red and Masson’s trichrome staining. The functional relevance between high mobility group box 1 (HMGB1) and inflammasome activation and the role of EGCG in it were analyzed by Western blotting. The effect of EGCG on autophagic flux was determined by Western blotting and flow cytometric analysis.

**Results:**

EGCG treatment efficiently was found to alleviate HBV-induced liver injury and fibrosis in a recombinant cccDNA (rcccDNA) chronic HBV mouse model, a proven suitable research platform for HBV-induced fibrosis. Mechanistically, EGCG was revealed to repress the activation of macrophage NLRP3 inflammasome, a critical trigger of HBV-induced liver fibrosis. Further study revealed that EGCG suppressed macrophage inflammasome through downregulating the level of extracellular HMGB1. Furthermore, our data demonstrated that EGCG treatment downregulated the levels of extracellular HMGB1 through activating autophagic degradation of cytoplasmic HMGB1 in hepatocytes. Accordingly, autophagy blockade was revealed to significantly reverse EGCG-mediated inhibition on extracellular HMGB1-activated macrophage inflammasome and thus suppress the therapeutic effect of EGCG on HBV-induced liver injury and fibrosis.

**Conclusion:**

EGCG ameliorates HBV-induced liver injury and fibrosis *via* autophagic degradation of cytoplasmic HMGB1 and the subsequent suppression of macrophage inflammasome activation. These data provided a new pathogenic mechanism for HBV-induced liver fibrosis involving the extracellular HMGB1-mediated macrophage inflammasome activation, and also suggested EGCG administration as a promising therapeutic strategy for this disease.

## 1 Introduction

Liver fibrosis is a reversible wound healing process characterized by the progressive accumulation of extracellular matrix (ECM) following liver injury, which may progress to irreversible cirrhosis and hepatocellular carcinoma without effective treatment (1, 2). It is now evident that prolonged liver injury leads to liver inflammation and subsequent fibrosis, in which HBV infection remains one of the leading causes. Besides the virus itself, the release of pro-inflammatory cytokines, especially IL-1β, plays a crucial role in promoting HBV-induced liver fibrosis (3, 4). Despite significant advances in the antiviral treatment of HBV infection, efficient therapies targeting HBV-induced chronic liver inflammation and fibrogenesis are still lacking (5). Therefore, therapeutic strategies that could inhibit virus-induced inflammatory responses, especially inflammasome activation, may be of great significance for treating HBV-induced liver fibrosis.

Accumulating evidence indicates that the activation of NLRP3 inflammasome in macrophages plays a vital role in driving chronic liver inflammation and the subsequent liver fibrosis (6–8). Of note, IL-1β or IL-18 may form a positive feedback loop with other inflammatory mediators, driving the amplification of inflammatory responses (9). Evidence indicates that, in the liver, inflammasome activation mainly happens in innate immune cells like macrophages, and to a much lesser extent in nonimmune cells including hepatocytes and hepatic stellate cells (HSCs) (6, 10). Reports have revealed the relevance of macrophage inflammasome activation to liver injury and chronic liver inflammation in conditions such as non-alcoholic steatohepatitis (NASH), alcoholic steatohepatitis and auto-immune hepatitis (AIH) (11–13); particularly, it is critical for the progression of liver fibrosis (14). Consistently, our previous investigation reported a crucial role of macrophage inflammasome/IL-1β signaling in the exacerbation of HBV-induced liver injury and fibrosis (15), suggesting that macrophage inflammasome may represent a promising therapeutic target against HBV-induced liver injury and fibrosis.

Green tea, one of the China’s greatest contributions to mankind, is considered to be the first Chinese herbal medicine (CHM) used by Chinese people since ancient times (16). Epigallocatechin-3-gallate (EGCG) is the most abundant and bioactive polyphenolic catechin in green tea (17). Studies indicate that EGCG possesses diverse physiological and pharmacological benefits, including anticarcinogenic, anti-oxidant, anti-inflammatory, and anti-microbial properties (18–20). We and others demonstrate that EGCG displays an inhibitory effect on HBV replication (21–23). Of interest, multiple clinical trials have confirmed the potential therapeutic effect of EGCG on chronic inflammatory diseases, including cystic fibrosis, osteoarthritis and atherosclerosis (24–26). It has been discovered that the effect of EGCG on inflammatory diseases is likely linked to the activation of autophagy (27). Autophagy is an evolutionally conserved process involving the lysosomal degradation of cytoplasmic contents, which mainly occurs under stress conditions like starvation (28). EGCG is reported to mimic the effects of dietary restriction, thereby inducing autophagic flux to degrade some pro-inflammatory agents, such as HMGB1 and AFP (27, 29). Of note, the activation of autophagic flux has been reported to efficiently alleviate liver injury and fibrosis (30, 31).

In the present study, our data showed that EGCG treatment alleviated HBV-induced liver injury and fibrosis in a recombinant cccDNA (rcccDNA) chronic HBV mouse model. Besides its inhibition of HBV replication, EGCG was found to repress macrophage NLRP3 inflammasome activation, a critical aggravator of liver fibrosis progression in mice. Further study revealed that EGCG downregulated extracellular HMGB1, a prototypic damage-associated molecular pattern molecule (DAMP), to inhibit macrophage NLRP3 inflammasome activation. Furthermore, EGCG treatment was found to suppress hepatocyte HMGB1 secretion in an autophagy-dependent manner, thereby decreasing extracellular HMGB1 and ameliorating liver injury and fibrogenesis.

## 2 Materials and methods

### 2.1 Animal experiments

The persistent recombinant cccDNA (rcccDNA) mouse model was established as described (15, 32). Briefly, albumin (Alb)-Cre transgenic mice (C57BL/6-Tg [Alb-Cre]), aged 6-8 weeks, were intravenously injected with 1.5 × 10^9^ plaque-forming units (PFU) of Ad/rcccDNA (OBIO Technology, Shanghai, China) in 200 μL of normal saline. 3-7 days later, sera HBsAg were detected to confirm the successful establishment of the rcccDNA mouse models. 30 weeks later, EGCG (25 mg/kg; Sigma) was injected intraperitoneally (i.p.) daily in rcccDNA mice for 42 consecutive days. To block autophagic flux, rcccDNA mice were i.p. daily injected with either saline or leupeptin (20 mg/kg; ApexBio, Houston, USA) 1 h prior to EGCG administration. For ATG5 knockdown *in vivo*, ATG5 siRNA (5’-CCATCAACCGGAAACTCAT-3’) (2 mg/kg) were premixed with *in vivo*-jet-PEI TM (Polyplus Transfection, Strasbourg, France) at a ratio specified by the manufacture and i.v. administrated in rcccDNA mice on the day before EGCG administration, which were subsequently applied twice a week for 42 days as described in our previous investigation (33). Animal experiments performed conformed to the Guide for the Care and Use of Medical Laboratory Animals (Ministry of Health, China) and were approved by the Animal Ethics Committee of Fudan University.

For the isolation of primary mouse hepatocytes and Kupffer cells, mouse livers were perfused with Hank’s balanced salt solution (HBSS) through the portal vein and subsequently with HBSS containing 0.025% collagenase IV. The digested liver was then dissected out thoroughly in collagenase buffer at 37°C for another 30 minutes. Then the suspension was filtered through a 70-μm cell strainer and centrifuged at 50 g for 3 min. The pellets, containing PMHs, were then washed with HBSS for three times and cultured with DMEM containing antibiotics and 10% FBS. The supernatants were centrifuged at 650 g for 7 min to collect the nonparenchymal cells, which were then re-suspended with HBSS and centrifuged on a density gradient solution of Percoll at 1000 g for 15 min. The KCs located in the interphase between the two density cushions were collected, washed with HBSS, and stained with PE-conjugated anti-F4/80 antibody coupled with anti-PE microbeads, and further isolated using a MACS system (Miltenyi Biotec) and characterized by flow cytometry **(** **Supplementary Figures 1A, B** **).**

### 2.2 qRT-PCR

qRT-PCR was performed as described previously (15, 34–36). Briefly, total RNA was purified from cells and liver tissues with TRIzol reagent (Takara, Dalian, China). according to the manufacturer’s protocol and reverse-transcribed into cDNA. Quantitative real-time PCR was performed using Roche Lightcycler 480 II and SYBR green system (Takara). GAPDH served as the endogenous control. The primer sequences used were provided in **Supplementary Table 1**.

### 2.3 Immunohistochemical staining, Sirius red and Masson’s trichrome staining

Liver tissues detached from mice were immediately fixed in 4% paraformaldehyde at room temperature. The fixed tissues were then embedded in paraffin wax, sectioned and then mounted on glass microscope slides. Sirius red and Masson’s trichrome staining of liver sections were performed by Service bio (Shanghai, China). In addition, the sections for α-SMA immunohistochemical staining were firstly deparaffinized and then rehydrated. After treated with 3% hydrogen peroxide and blocked with 5% bovine serum albumin, the sections were then incubated sequentially with primary antibody against α-SMA (**Supporting Table S2**), biotin-labeled secondary antibody and avidin-biotin complex (ABC). Peroxidase staining was developed with 3,3’-diaminobenzidine (DAB) solution and counterstained with hematoxylin. Microscopy images were achieved by a Nikon Eclipse Ts2 microscope and analyzed with the Image Pro-plus software.

### 2.4 Cell culture, transfection and HBV infection

Human hepatoma cells Huh7, HepAD38 cells (in which the HBV replication is under the control of a tetracycline-regulated promoter) were cultured in Dulbecco’s modified Eagle’s medium (DMEM) supplemented with 10% fetal bovine serum, 100 μg/mL penicillin and 100 μg/mL streptomycin in a 37 °C incubator containing 5% CO_2_. Primary human hepatocytes (PHHs) were isolated from liver resections as previously described (15, 34, 36). The human liver resections were received from the Department of Liver Surgery and Transplantation of Zhongshan Hospital (Fudan University, Shanghai, China). PHHs were seeded into collagen-I-coated culture plates with Williams E medium containing 5% FCS, 100 μg/mL penicillin, 100 μg/mL streptomycin, 5 μg/mL insulin, 5 × 10^-5^ M hydrocortisone and 2% DMSO. Replication-competent plasmid (pHBV1.3) were transfected into Huh7 cells using Hieff Trans™ Liposomal Transfection reagent (Yeasen, Shanghai, China) according to the manufacturer’s instructions. SiRNAs targeting human ATG5 (5’-CAUCUGAGCUACCCGGAUA-3’) were electrotransfected into HepAD38 cells using Amaxa Nucleofector (Amaxa GmbH, Cologne, Germany). For HBV infection experiments, PHHs were infected with different doses of HBV particles (MOI = 50/100/200/400) in the presence of 4% polyethylene glycol (PEG) 8000. Approximately 16 hours after infection, cells were washed three times with phosphate-buffered saline (PBS) and then maintained in medium supplemented with 2.5% dimethyl sulfoxide (DMSO). In all transfection assays, β-galactosidase expression plasmid (pCMV-β-gal) was cotransfected to normalize the transfection efficiency.

### 2.5 Cell viability by CCK8 assay

Cells were seeded in a 96-well plate in a volume of 100 μL and maintained at 37 °C in an incubator containing 5% CO_2_. To determine the cell viability, 20 μL of CCK-8 solution (Dojindo Laboratories, Kumamoto, Japan) was added to each well and co-incubated with cells for 2 h. The absorbance of each well was then assessed at 450 nm by a microplate reader (GENios Tecan). The data were expressed as a percentage relative to those obtained in the control groups.

### 2.6 ELISA for HMGB1 and cytokines

Levels of HMGB1 in serum or supernatant were determined by ELISA kits (Shino-Test, Sagamihara-shi, Kanagawa, Japan). Levels of IL-1β and IL-18 in serum or supernatants were analyzed with the corresponding ELISA kits (eBioscience. CA, USA) following the manufacturers’ instructions.

### 2.7 Western blotting

Western blotting was performed as described previously (34–36). Cytoplasmic and nucleic extracts of cells or liver tissues were separated using the protein extraction kit (Beyotime, Shanghai, China) following the manufacturer’s protocols. Intracellular proteins in cells or liver tissues were extracted using cold lysis buffer (25 mM Tris-HCl, PH 7.6, 150 mM NaCl, 1% NP-40, 0.1% SDS) plus protease inhibitor cocktail and phenylmethanesulfonyl fluoride (PMSF) on ice. The supernatant proteins were concentrated from the cultured media using Amicon Ultra Centrifugal Filter Devices (Millipore, USA) according to the manufacturer’s instructions. The collected protein samples were subjected to sodium dodecyl sulfate-polyacrylamide gel electrophoresis (SDS-PAGE) and transferred onto PVDF membranes (Millipore). Membranes for supernatant proteins were stained with Ponceau S (Sigma-Aldrich) as the loading control. Subsequently, membranes were blocked for 1 h with nonfat dry milk solution (5% in TBS) containing 0.1% Tween-20. The blots were probed subsequently with the indicated primary antibodies and the corresponding secondary antibodies. Immunoreactive bands were visualized by enhanced chemiluminescence (Yeasen, Shanghai, China). The antibodies used in this study were listed in **Supplementary Table 2**.

### 2.8 Caspase-1 activity assay

The caspase-1 activity in mouse liver tissues and culture cell lysates was determined by a caspase-1 activity assay kit **(**Beyotime, Shanghai, China). For the determination of caspase-1 activity in liver tissues, the liver tissues were first homogenized, and centrifuged at 16,000 g for 15 min at 4°C. The supernatants were then collected and added with a substrate of caspase-1 (Ac-YVAD-pNA), and then incubated for 60-120 min at 37°C. When the solution showed an obvious yellow p-nitroaniline (pNA) colour, we stopped the reaction and tested the sample at 405 nm using a microplate reader (GENios Tecan). Data were quantified by a standard curve. For the determination of caspase-1 activity in cell lysates, the indicated cells were lysed on ice for 15 min and then centrifuged at 16,000 g for 15 min at 4°C. The supernatant was collected, and the following steps were the same as above.

### 2.9 Flow cytometric analysis

Flow cytometric analysis for LC3-II and p62 was described as in our previous investigation (15, 22). Briefly, for the detection of LC3-II, single cell suspensions were first harvested with trypsin treatment. Cells were then washed with PBS containing 0.05% saponin and incubated with rabbit anti-LC3 antibody for 20 min. Subsequently, cells were incubated with anti-rabbit FITC secondary antibody for another 20 min. As for p62 detection, cells were treated with fixation and permeabilization buffer, followed by incubation with mouse anti-p62 antibody, and subsequently with anti-mouse FITC secondary antibody. Data were obtained by a BD FACS Calibur (BD Biosciences) in CellQuest (BD Biosciences) and analyzed using FlowJo software (Tree Star).

### 2.10 Statistical analysis

Data were commonly expressed as the mean ± SEM unless specific statement. Statistical significance was determined using a two-tailed, unpaired Student’s t-test (for two sample comparisons) or one-way analysis of variance (ANOVA) (for multiple comparisons). Pearson’s correlation coefficient was used to evaluate the quality of linear correlations. The statistical analyses were performed using GraphPad Prism software version 8.0 (GraphPad Software, La Jolla, CA). Differences were considered statistically significant at *P* < 0.05.

## 3 Results

### 3.1 EGCG treatment ameliorates HBV-induced liver injury and fibrosis

**Figure 1:**
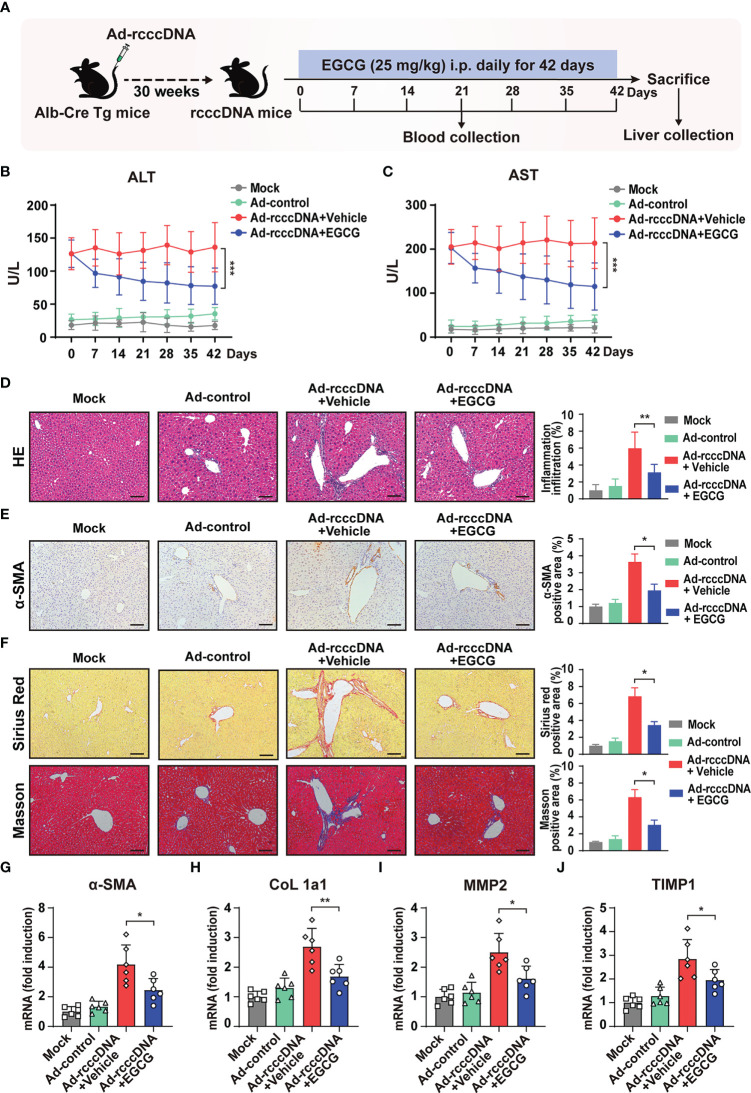
EGCG treatment alleviates HBV-induced liver injury and fibrosis. **(A)** Alb-Cre Tg mice were intravenously injected with Ad/rcccDNA at 1.5 × 10^9^ PFU to establish an HBV-induced liver fibrosis mouse model, and the rcccDNA mice were then received an intraperitoneal injection of 25 mg/kg EGCG daily for 42 days (*n* = 6 per group). **(B, C)** Levels of serum ALT and AST were determined by ELISA. **(D)** Liver inflammation infiltration was detected by hematoxylin and eosin **(H, E)** staining. **(E, F)** α-SMA expression was determined by immunohistochemical staining with DAB (brown), and liver collagen deposition was determined by Sirius red staining and Masson’s trichrome staining. Quantification of α-SMA-, Sirius red- and Masson- positive areas were measured in 5 random fields of each slide using Image Pro-plus software. Scale bar: 100 μm. **(G–J)** The mRNA expression levels of α-SMA, CoL 1a1, MMP2 and TIMP were determined by qRT-PCR (normalized to GAPDH). Data are shown as mean ± SEM and compared by two-way analysis of variance (ANOVA) or unpaired Student’s t test. *P < 0.05, **P < 0.01, ***P < 0.001.

To investigate the effect of EGCG on HBV-induced liver injury and fibrosis, we intravenously injected 1.5×10^9^ PFU of adenovirus carrying recombinant HBV cccDNA (Ad/rcccDNA) into the Alb-Cre Tg mice to establish a chronic rcccDNA mouse model, which has been demonstrated to be a suitable platform to investigate the HBV-induced fibrosis (15, 32). The rcccDNA mice were then treated with EGCG (25 mg/kg) daily for 42 consecutive days through intraperitoneal (i.p.) injection as shown in **Figure 1A**. The dose of EGCG was chosen according to the results of our dose-response experiments, which indicated that 25 mg/kg of EGCG was well-tolerated and non-toxic in rcccDNA mice **(** **Supplementary Figures 2A, B** **)**. Our results showed that, upon EGCG treatment, the levels of serum alanine aminotransferase (ALT) and aspartate transaminase (AST), key markers of liver injury, in the rcccDNA mice were significantly downregulated **(** **Figures 1B, C** **)**. Further, results from hematoxylin and eosin (H&E) staining showed that EGCG treatment could suppress inflammatory cell infiltration in liver tissues of rcccDNA mice **(** **Figure 1D** **)**. Furthermore, EGCG treatment was found to suppress the fibrosis progression efficiently, as demonstrated by the decreased levels of alpha-smooth muscle actin (α-SMA) and the diminished collagen deposition in portal areas of the rcccDNA mouse liver **(** **Figures 1E, F** **)**. Consistent with the histological analysis, the qRT-PCR data showed that EGCG treatment significantly downregulated the mRNA expression levels of fibrosis-related genes including α-SMA, alpha-1 type1 collagen (Col 1a1), matrix metallopeptidase 2 (MMP2) and tissue inhibitor of metalloproteinases-1 (TIMP1) in the liver tissue of this chronic rcccDNA mouse model **(** **Figures 1G–J** **)**.

Taken together, the above results indicated that EGCG treatment could attenuate the severity of the liver injury and impede the progression of HBV-induced liver fibrosis in rcccDNA mouse model.

### 3.2 EGCG repress the activation of macrophage inflammasome, a critical aggravator of HBV-induced liver injury and fibrosis

**Figure 2:**
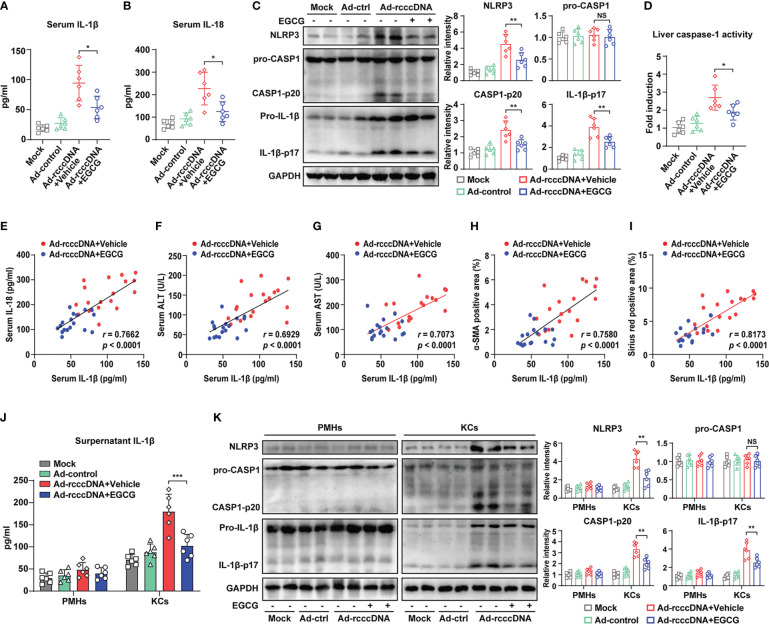
EGCG suppresses the macrophage NLRP3 inflammasome activation in HBV-induced liver injury and fibrosis mouse model. **(A, B)** IL-1β and IL-18 levels in the sera of mock mice, Ad-control mice and rcccDNA mice treated with or without EGCG (*n* = 6) were determined by ELISA. **(C)** Protein levels of NLRP3, pro-caspase-1, caspase-1 p20, pro-IL-1β and IL-1β p17 in liver tissues were determined by Western blotting. Relative levels of NLRP3, pro-caspase-1, caspase-1 p20 and IL-1β p17 were determined by densitometric analysis, and the value from control group was set at 1.0. **(D)** Liver caspase-1 activity of liver tissues was determined by caspase-1 activity assay. **(E)** Correlation analysis of serum IL-1β with serum IL-18. **(F–I)** The correlations of serum IL-1β with serum ALT, serum AST, liver α-SMA positive area, and liver Sirius red positive area in rcccDNA mice treated with or without EGCG. **(J, K)** PMHs and KCs were isolated from mock mice, Ad-control mice and rcccDNA mice treated with or without EGCG, followed by the determination of NLRP3 inflammasome activation in PMHs and KCs (*n* = 6). Supernatant IL-1β levels were determined by ELISA **(J)**. Protein expression levels of NLRP3, pro-caspase-1, cleaved caspase-1 p20, pro-IL-1β, and cleaved IL-1β p17 were determined by Western blotting as in C **(K)**. Data are shown as means ± SEM and compared by unpaired Student’s t-test. *P < 0.05, **P < 0.01, ***P < 0.001. NS, no significance.

Accumulating evidence indicates that besides the virus itself, the release of pro-inflammatory cytokines, especially IL-1β, may play a more crucial role in promoting HBV-induced liver fibrosis (37, 38). Our previous investigation also demonstrated that macrophage inflammasome/IL-1β signaling was critical for the development of HBV-induced liver injury and fibrosis in rcccDNA persistent mice (15). Considering the possible anti-inflammatory properties of EGCG, we therefore investigated the effect of EGCG treatment on inflammasome activation in rcccDNA mice. Our data showed that EGCG treatment significantly inhibited NLRP3 inflammasome activation in rcccDNA mice, as demonstrated by the downregulated IL-1β and IL-18 levels in mice sera **(** **Figures 2A, B** **)**, and the decreased levels of caspase-1 activity, NLRP3, caspase-1 p20 and IL-1β-p17 proteins in the liver tissues **(** **Figure 2C** **)**, and the decreased liver caspase-1 activity **(** **Figure 2D** **)**. Correlation analysis revealed a positive relationship of the decreased serum IL-1β levels with decreased serum IL-18 levels in rcccDNA mice upon treatment by EGCG **(** **Figure 2E** **)**. Further, we observed a positive relationship of the decreased serum IL-1β levels with the decreased markers of liver injury, including serum ALT and serum AST **(** **Figures 2F, G** **)**, and the decreased markers of fibrosis, including α-SMA or Sirius red positive area, in EGCG-treated rcccDNA mice **(** **Figures 2H, I**), further supporting the critical role of inflammasome signaling in EGCG-mediated inhibition of liver injury and fibrosis. To determine which cell type was mainly responsible for EGCG-mediated inhibition of inflammatory response in rcccDNA mice, we separated primary mouse hepatocytes (PMHs) and mouse liver macrophages (Kupffer cells, KCs) from the indicated mice, and then tested the effect of EGCG on the NLRP3 inflammasome activation. Consistent with our previous findings, KCs, rather than hepatocytes, were found to be mainly responsible for the inflammasome activation in rcccDNA mice; however, the inflammasome activation could be significantly inhibited by EGCG, as demonstrated by the decreased secretion of IL-1β (**Figure 2J**) and IL-18 (**Supplementary Figure 3A**), the decreased protein levels of NLRP3, caspase-1 p20, IL-1β-p17 (**Figure 2K**) as well as the decreased caspase -1 activity (**Supplementary Figure 3B**).

Together, our data indicated that EGCG alleviated HBV-induced liver injury and fibrosis mainly through inhibiting inflammasome activation in KCs rather than in hepatocytes in rcccDNA mice.

### 3.3 EGCG inhibited macrophage inflammasome in HBV-induced liver fibrosis *via* downregulation of extracellular HMGB1

**Figure 3:**
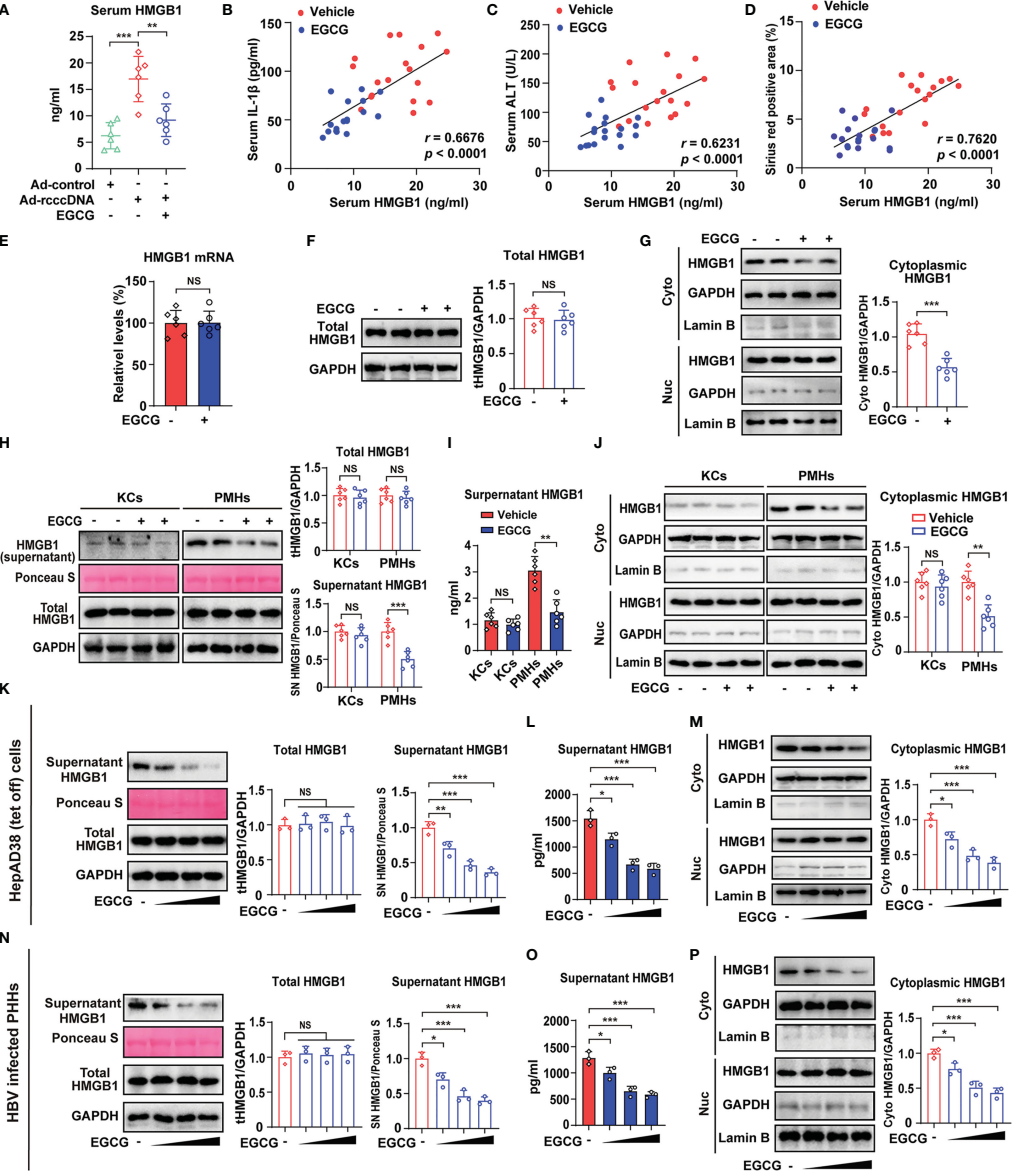
EGCG degrades cytoplasmic HMGB1 and reduces its following secretion from HBV-infected hepatocytes. **(A–G)** RcccDNA mice were treated with or without 25 mg/kg EGCG daily for 42 days (*n* = 6). Serum HMGB1 levels were examined by ELISA **(A)**. Correlations of serum HMGB1 with serum IL-1β **(B)**, serum ALT **(C)** and Sirius red-positive areas **(D)** in liver tissues of rcccDNA mice treated with or without EGCG. Hepatic HMGB1 mRNA levels were determined by qRT-PCR **(E)**. Hepatic HMGB1 protein levels **(F)** and HMGB1 protein levels in cytoplasmic and nucleic extracts of liver tissues **(G)** were determined by Western blotting. Relative HMGB1 protein levels were determined by densitometric analysis, and the value from the control group was set at 1.0. **(H–J)** PMHs and KCs were isolated from rcccDNA mice treated with or without EGCG (*n* = 6). Protein levels of HMGB1 in supernatant and cell lysate were determined by Western blotting as in F **(H)**. Supernatant HMGB1 levels were determined by ELISA **(I)**. HMGB1 protein levels in cytoplasmic and nucleic extracts of PMHs and KCs were determined by Western blotting as in F **(J)**. **(K–M)** HepAD38 cells (Tet off) were treated with increasing dosage of EGCG (12.5, 25 and 50 μM) for 24 hours, followed by the determination of HMGB1 secretion, intracellular and cytoplasmic HMGB1 as in **(F, N–P)** HBV-infected PHHs were treated with increasing dosage of EGCG (12.5, 25 and 50 μM) for 24 hours, followed by the determination of HMGB1 secretion, intracellular and cytoplasmic HMGB1 as in **(F)** Data are shown as mean ± SEM and compared by unpaired Student’s t test or one-way analysis of variance (ANOVA). *P < 0.05, **P < 0.01, ***P < 0.001. NS, no significance.

High mobility group box 1 (HMGB1), a highly conserved nuclear DNA-binding protein that stabilizes nucleosomes and regulates gene expression, will be sensed as an endogenous danger signal by the immune system when it occurs at extracellular milieu and thus contributes to the development of inflammatory diseases (39, 40). We and others have demonstrated that extracellular HMGB1 may serve as an alarmin signal to activate macrophage inflammatory responses (41, 42), and a key driver of inflammasome activation and liver fibrosis (43). We had therefore detected the level of serum HMGB1 and the effect of EGCG on it. It was found that, compared with that in Ad-control mice, the level of serum HMGB1 in rcccDNA mice was significantly upregulated, which could be significantly attenuated by the treatment of EGCG (**Figure 3A**). Of note, the downregulation of HMGB1 was closely correlated with the decreased serum levels of IL-1β, serum ALT levels and hepatic Sirius red positive area in rcccDNA mice (**Figures 3B–D**). Further study showed that EGCG treatment did not affect HMGB1 expression (**Figures 3E, F**), but decreased the cytoplasmic levels of HMGB1 protein in the liver tissues of rcccDNA mice (**Figure 3G**). Furthermore, we determined the cellular target for EGCG-mediated downregulation of HMGB1 by isolating hepatocytes and KCs from indicated mice. It was found that EGCG significantly downregulated the supernatant and cytoplasmic HMGB1 in PMHs, but not in KCs (**Figures 3H–J**), and the decreased supernatant and cytoplasmic HMGB1 in PMHs appeared to positively correlate with the attenuation of HBV-induced fibrosis (**Supplementary Figures 4A–H**). Consistent with the *in vivo* data, further study revealed that EGCG significantly downregulated the secretion of HMGB1 (**Figures 3K, L**), while having no significant effect on its intracellular expression (**Figure 3K**) in HBV-replicating hepatoma cells (HepAD38 cells); further, EGCG was revealed to efficiently downregulate the cytoplasmic HMGB1 in HepAD38 cells (**Figure 3M**). Similar phenomena were further confirmed in HBV-infected PHHs (**Figures 3N–P** **)**.

Together, EGCG treatment could decrease the cytoplasmic HMGB1 proteins in HBV-infected hepatocytes and reduce its following secretion.

### 3.4 EGCG promotes the degradation of HMGB1 through activating autophagic flux in hepatocytes

**Figure 4:**
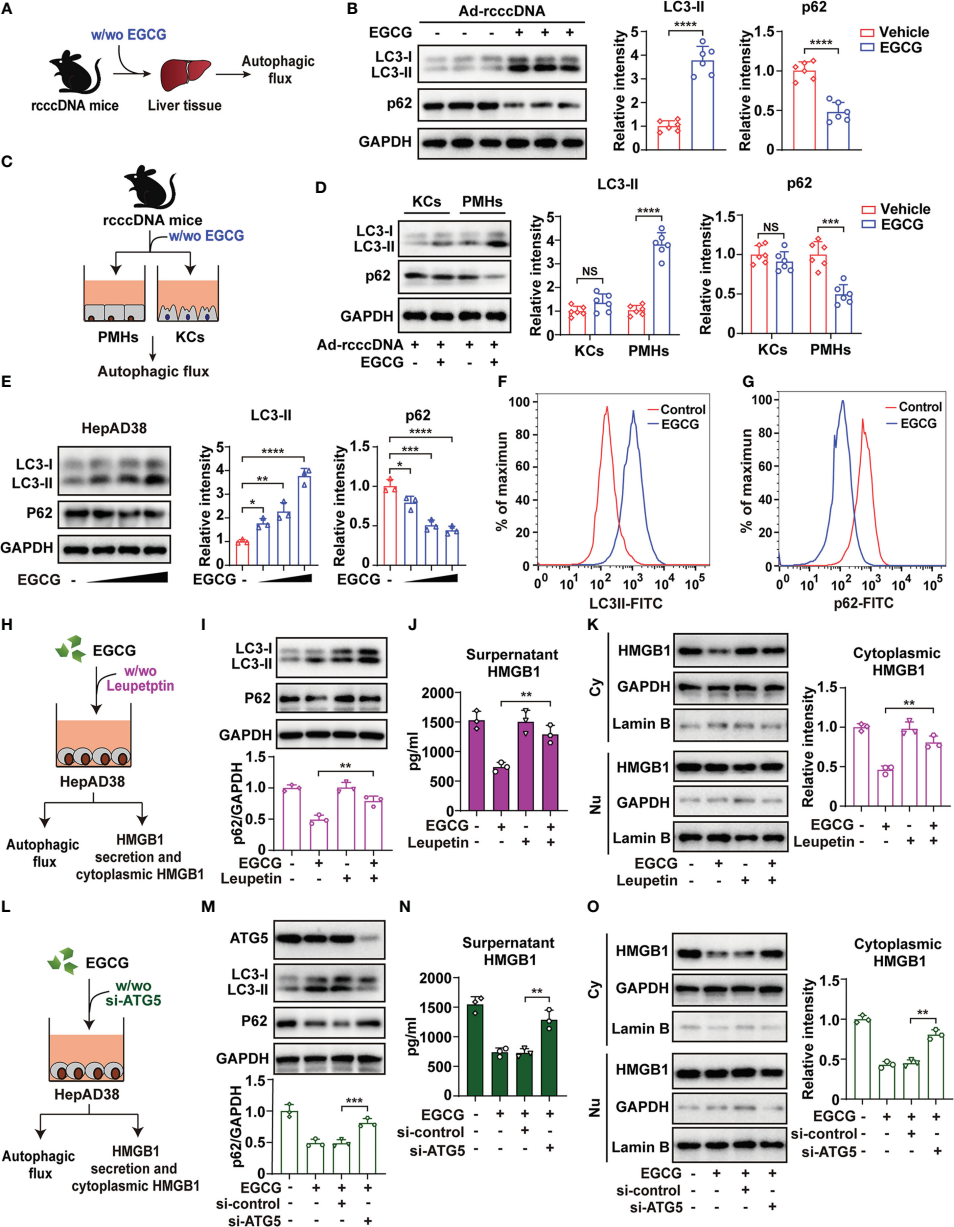
EGCG promotes the degradation of cytoplasmic HMGB1 through activating autophagic flux in hepatocytes. **(A)** Schematic experimental setup: rcccDNA mice were treated with or without 25 mg/kg EGCG daily for 42 days, followed by determination of autophagic flux in liver tissues (n = 6). **(B)** Protein levels of LC3-II and p62 were determined by Western blotting. Right panel: relative protein levels of LC3-II and p62 were determined by densitometric analysis, and the value from rcccDNA mice without EGCG treatment was set at 1.0. **(C)** Schematic experimental setup: PMHs and KCs were isolated from rcccDNA mice treated with or without EGCG for the determination of autophagic flux in cell lysates, respectively (n = 6). **(D)** The protein levels of LC3-II and p62 were determined by Western blotting as in **(B, E)** HepAD38/Tet off cells were treated with increasing doses of EGCG (12.5, 25 and 50 μM) for 24 h, followed by examining the protein levels of LC3-II and p62 by Western blotting as in **(B, F, G)** HepAD38/Tet off cells were treated with 25 μM of EGCG for 24 hours, followed by detecting the levels of LC3-II **(F)** and p62 **(G)** by FACS. **(H)** Schematic illustration of the effect of blocking autophagic flux by leupeptin on cytoplasmic HMGB1 and its following secretion from HBV replicating cells. **(I, J)** HepAD38 cells were treated with 25 μM EGCG for 24 h in the presence of 200 μg/mL of leupeptin, and the protein levels of LC3-II and p62 were determined by Western blotting as in B **(I)**. Supernatants HMGB1 levels **(J)** and HMGB1 protein levels in cytoplasmic and nucleic extracts of HepAD38 cells **(K)** were determined by ELISA and Western blotting, respectively. **(L–O)** Schematic illustration of the effect of blocking autophagic flux by si-ATG5 on cytoplasmic HMGB1 and its following secretion from HBV replicating cells **(L)**. HepAD38 cells were first transfected with si-ATG5 for 48 h, followed by 25 μM EGCG treatment for another 24 h. Protein levels of LC3-II and p62 **(M)**, supernatant HMGB1 **(N)** and HMGB1 protein levels in cytoplasmic and nucleic extracts **(O)** were determined as in I to **(K)** All data are shown as mean ± SEM and compared by unpaired Student’s t test or one-way analysis of variance (ANOVA). *P < 0.05, **P < 0.01, ***P < 0.001, ****P < 0.0001. NS, no significance.

Autophagy, a lysosome-dependent degradation system, represents a therapeutic target for various liver diseases (28). We and others have demonstrated EGCG as an important autophagy inducer (22, 44). We therefore explore whether the degradation of cytoplasmic HMGB1 is attributed to the autophagic flux induced by EGCG (**Figure 4A**). Expectedly, EGCG treatment activated autophagic flux in the liver tissue of rcccDNA mice, as demonstrated by the increased levels of LC3-II, a well-established marker for autophagy induction, and decreased protein levels of p62, a widely used indicator of autophagic flux (**Figure 4B**). Further, we isolated PMHs and KCs from rcccDNA mice treated with or without EGCG, and then determined the autophagic flux in these cells (**Figure 4C**). It was found that EGCG treatment significantly upregulated LC3-II and downregulated p62 protein levels in PMHs, but not in KCs (**Figure 4D**), indicating EGCG-mediated autophagic flux mainly occurred in hepatocytes of rcccDNA mice. Additionally, the extent of autophagic flux in PMHs appeared to correlate with EGCG-mediated attenuation of HBV-induced liver injury and fibrosis (**Supplementary Figures 5A–J**).

Further, we treated HBV-replicating HepAD38 cells with increasing doses of EGCG, followed by examining the levels of LC3-II and p62. As expected, EGCG was found to dose-dependently increase LC3-II and decrease p62 protein levels, indicating that EGCG induced autophagic flux in HBV-replicating HepAD38 cells (**Figure 4E**). Fluorescence-activated cell sorting (FACS) analysis was used to further confirm this finding (**Figures 4F, G**). Similar data were also obtained in pHBV1.3-transfected Huh7 cells (**Supplementary Figures 6A, B**), suggesting that EGCG could induce autophagic flux in HBV-replicating cells, even though HBV *per se* was reported to actively interrupt autophagic flux (45). To investigate the effect of EGCG-mediated autophagic flux on HMGB1 secretion and its cytoplasmic protein levels, we treated cells with leupeptin, a well-established inhibitor for autophagic flux (46) (**Figure 4H**). Our results showed that leupeptin significantly blocked the autophagic flux induced by EGCG in HepAD38 cells; however, once autophagic flux was blocked, EGCG-mediated inhibition of HMGB1 secretion and degradation of cytoplasmic HMGB1 was significantly reversed (**Figures 4I–K**). We further investigated the effect of autophagy flux on EGCG-mediated inhibition of HMGB1 secretion and its cytoplasmic protein levels by knockdown of autophagy related gene 5 (ATG5), an essential molecule for autophagy induction (**Figure 4L**), and similar data as those from leupeptin treatment were obtained (**Figures 4M–O**).

Together, our results demonstrated that EGCG promoted the degradation of cytoplasmic HMGB1 *via* inducing autophagic flux, and thus inhibited the following HMGB1 secretion from hepatocytes.

### 3.5 Blockage of autophagic flux reverses EGCG-mediated downregulation of HMGB1 secretion and the following macrophage NLRP3 inflammasome activation

**Figure 5:**
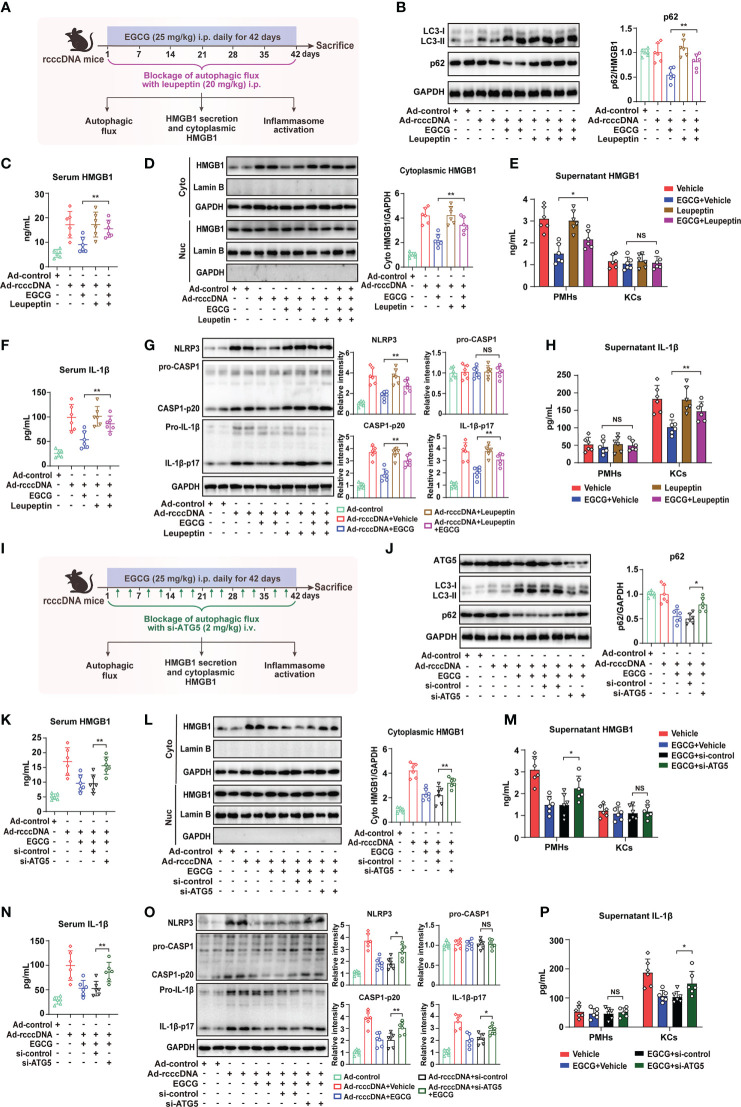
Blockage of autophagic flux reverses EGCG-mediated inhibition on HMGB1 secretion from hepatocytes and the subsequent macrophage NLRP3 inflammasome activation. **(A–H)** Schematic experimental setup: rcccDNA mice were treated with EGCG (25 mg/kg) daily for 42 days through i.p. injection in the presence of leupeptin (20 mg/kg), followed by the determination of autophagic flux, HMGB1 secretion and inflammasome activation (n = 6) **(A)**. Protein levels of LC3-II and p62 in liver tissues were determined by Western blotting. *Right panel:* relative protein level of p62 was determined by densitometric analysis, and the value from control group was set at 1.0 **(B)**. Serum HMGB1 levels were determined by ELISA **(C)**. HMGB1 protein levels in cytoplasmic and nucleic extracts of liver tissues were determined by Western blotting as in B **(D)**. HMGB1 protein levels in the supernatants of PMHs or KCs were determined by ELISA **(E)**. Serum IL-1β levels were determined by ELISA **(F)**. Protein levels of NLRP3, pro-caspase-1, cleaved caspase-1 p20, pro-IL-1β and cleaved IL-1β p17 in liver tissues were determined by Western blotting as in B **(G)**. Supernatants IL-1β levels of PMHs or KCs were determined by ELISA **(H)**. **(I–P)** Schematic experimental setup: rcccDNA mice were i.p. injected with EGCG (25 mg/kg) for 42 days, in which si-ATG5 (2 mg/kg) were i.v. injected twice a week to block the autophagic flux through tail vein, followed by the determination of autophagic flux, HMGB1 secretion and inflammasome activation (n = 6) **(I)**. Protein levels of ATG5, LC3-II and p62 **(J)**, serum HMGB1 levels **(K)**, HMGB1 protein levels in cytoplasmic and nucleic extracts of liver tissues **(L)**, HMGB1 protein levels in the supernatants of PMHs or KCs **(M)**, serum IL-1β levels **(N)**, protein levels of NLRP3, pro-caspase-1, cleaved caspase-1 p20, pro-IL-1β and cleaved IL-1β p17 in liver tissues **(O)** and IL-1β levels in the supernatants of PMHs or KCs **(P)** were determined as in B to **(H)** All data are shown as mean ± SEM and compared by unpaired Student’s t test. *P < 0.05, **P < 0.01. NS, no significance.

As the blockage of autophagic flux significantly reversed the inhibitory effect of EGCG on HMGB1 secretion *in vitro*, we further investigated whether the blockage of autophagic flux using leupeptin could reverse the EGCG-mediated inhibition of HMGB1 secretion and the subsequent NLRP3 inflammasome activation in rcccDNA mice **(** **Figure 5A** **)**. Our results showed that, upon treatment with leupeptin, EGCG-mediated autophagic flux in rcccDNA mice was efficiently repressed, as demonstrated by the upregulation of p62 protein **(** **Figure 5B**). Of note, it was found that blocking autophagic flux by leupeptin significantly reversed the EGCG-mediated inhibition of HMGB1 release, as revealed by the upregulation of serum HMGB1 levels (**Figure 5C**), cytoplasmic HMGB1 levels in liver tissue (**Figure 5D**) as well as the HMGB1 levels in the culture supernatants of PMHs (**Figure 5E**). Consistent with its effect on HMGB1 secretion, blockage of autophagic flux by leupeptin effectively abrogated the repressive effect of EGCG on NLRP3 inflammasome activation, as indicated by the upregulated serum levels of IL-1β (**Figure 5F**) and the upregulated protein levels of NLRP3, caspase-1 p20, and IL-1β-p17 in liver tissues (**Figure 5G**) as well as the upregulation of IL-1β levels in the culture supernatants of KCs (**Figure 5H**). To further confirm the impact of autophagy blockage on EGCG-mediated inhibition of HMGB1 secretion and NLRP3 inflammasome activation, we knocked down the expression of ATG5 in rcccDNA mice (**Figure 5I**). As expected, similar data as those from leupeptin treatment were obtained (**Figures 5J–P**).

It is reported that HBV, *per se*, might be directly involved in the pathogenesis of liver fibrosis. We thus investigated whether blockage of autophagic flux could impact EGCG-mediated inhibition of HBV replication. Consistent with previous investigation (21), EGCG treatment could efficiently inhibit HBV replication (**Supplementary Figures 7A, B** **)**, however blockage of autophagic flux with either leupeptin or siATG5 did not have a significant effect on EGCG-mediated inhibition of HBV replication (**Supplementary Figures 7C, D** **)**, further indicating the “specific” effect of EGCG- mediated autophagy on HMGB1 degradation.

Together, these data indicated that blockage of autophagic flux using leupeptin or si-ATG5 could reverse the inhibitory effect of EGCG on HMGB1 secretion and the subsequent NLRP3 inflammasome activation in rcccDNA mice.

### 3.6 Blockage of autophagic flux reverses EGCG-mediated amelioration of HBV-induced liver injury and fibrosis

**Figure 6:**
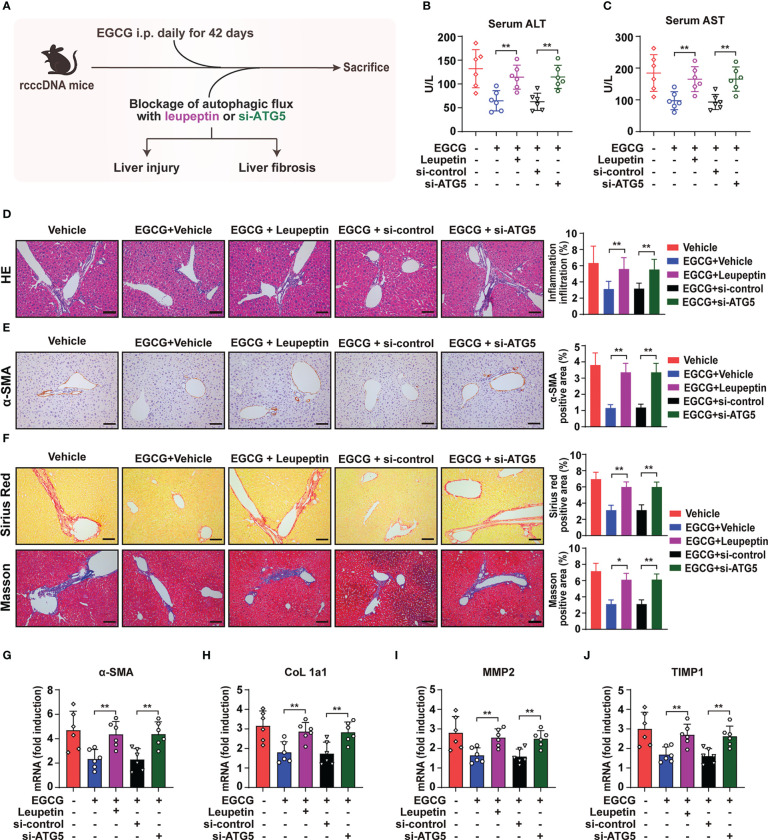
Blockage of autophagic flux reverses the protective effect of EGCG on HBV-induced liver injury and fibrosis. **(A)** Schematic illustration of the effect of blockage of autophagic flux using leupeptin and si-ATG5 on EGCG-mediated inhibitory effect on HBV-induced liver injury and liver fibrosis in rcccDNA mice (n = 6). **(B, C)** Levels of ALT **(B)** and AST **(C)** in mice sera were determined by the ELISA. **(D)** Liver inflammation infiltration was determined by Hematoxylin and eosin **(H, E)** staining. **(E)**α-SMA expression level was determined by immunohistochemical staining with DAB (brown). **(F)** liver collagen deposition was determined by Sirius red staining and Masson’s trichrome staining. Quantification of liver inflammation infiltration, α-SMA-, Sirius red- and Masson- positive areas were measured in 5 random fields of each slide using Image Pro-plus software. Scale bar: 100μm. **(G–J)** The mRNA expression levels of α-SMA **(G)**, CoL 1a1 **(H)**, MMP2 **(I)** and TIMP1 **(J)** were determined by qRT-PCR (normalized to GAPDH). Data are shown as mean ± SEM and compared by unpaired Student’s t test or one-way analysis of variance (ANOVA). *P < 0.05, **P < 0.01.

Given that blockage of autophagic flux could reverse EGCG-mediated suppression on HMGB1 secretion and NLRP3 inflammasome activation, we thus further blocked the hepatic autophagic flux in rcccDNA mice by leupeptin and si-ATG5 using the same method as in **Figure 6**, and then tested the effect of EGCG on HBV-induced liver injury and fibrosis (**Figure 6A**). Our results showed that blocking autophagic flux with leupeptin or si-ATG5 significantly reversed the protective effect of EGCG on liver injury, as demonstrated by the upregulated levels of serum ALT (**Figure 6B**), serum AST (**Figure 6C**) and liver inflammatory infiltration (**Figure 6D**). Consistently, the curative effect of EGCG on liver fibrosis was reversed by leupeptin and si-ATG5 treatment, as evidenced by the increased protein expression level of fibrosis-related gene α-SMA (**Figure 6E**) and increased collagen deposition (**Figure 6F**) in the liver tissues of rcccDNA mice. Additionally, qRT-PCR analysis revealed that leupeptin or si-ATG5 treatment could reverse the EGCG-mediated downregulation of the mRNA levels of fibrosis-related genes, including α-SMA, CoL 1a1, MMP2 and TIMP1, in the liver tissues of rcccDNA mice (**Figures 6G–J**).

Together, in agreement with the effect of leupeptin and si-ATG5 on EGCG-mediated inhibition of HMGB1 secretion and NLRP3 inflammasome activation as shown in **Figure 6**, blockage of autophagic flux did reverse the inhibitory effect of EGCG on HBV-induced liver injury and fibrosis.

## 4 Discussion

Liver fibrosis is a reversible wound-healing process in response to persistent liver injury, which could progress to irreversible cirrhosis and hepatocellular carcinoma without proper treatment (1, 2). HBV infection remains one of the major causes of liver fibrosis around the world. Although HBV *per se* has been found to have direct effect on promoting fibrogenesis (47), accumulating evidence demonstrates that HBV-induced immune response may play a more crucial role in the pathogenesis of liver fibrosis (3, 4).

Here, we discovered that EGCG, the most abundant bioactive catechin in green tea (17), efficiently attenuated HBV-induced liver injury and fibrosis. Mechanistically, besides its inhibition of HBV, EGCG suppressed the macrophage inflammasome activation, a key aggravator of HBV-induced liver fibrosis. Further, our findings revealed that EGCG suppressed macrophage inflammasome activation *via* autophagic degradation of HMGB1, a prototypic DAMP, in HBV-infected hepatocytes and thus alleviated HBV-induced liver injury and fibrosis.

Due to the lack of HBV entry receptor in mice, the investigation of effective therapies for HBV-associated inflammatory liver diseases has been hampered by the lack of suitable mouse models (48). A recent study found that in a recombinant cccDNA (rcccDNA) mouse model, a sustained necroinflammatory response and fibrosis were observed in the liver, indicating this mouse model may act as a suitable platform to investigate strategies against CHB and HBV-induced liver fibrosis (32), which was further confirmed in our previous investigation (15). Using this model, our present data suggested EGCG as a protective agent against HBV-induced liver injury and fibrosis.

Increasing evidence demonstrate that virus-induced inflammatory response may play a more critical role in HBV-induced liver fibrosis (14, 15), in which inflammasome activation receives particular attention. Of note, the activation of inflammasome-IL-1β signaling may induce a positive feedback loop between pro-inflammatory cytokines, which would perpetuate inflammation and subsequently promote the progression of fibrosis (9). Accumulating evidence suggests macrophage as the primary source of pro-inflammatory cytokines in the liver, supporting its role in hepatic injury and fibrosis (9, 49). Previously, we had also reported that STING signaling activation could alleviate HBV-induced liver injury and fibrosis *via* targeting macrophage inflammasome activation (15). Our present data further confirmed that, in rcccDNA mice, hepatic inflammasome activation happened mainly in KCs, not PMHs; while EGCG treatment was found to significantly reversed these phenomena, suggesting that inhibition of macrophage NLRP3 inflammasome might be the underlying mechanism by which EGCG alleviated HBV-induced liver injury and fibrosis. Similarly, inhibiting macrophage NLRP3 inflammasome activation could reduce liver injury and fibrosis of other etiologies. However, the molecular mechanisms for EGCG-mediated inhibition of macrophage inflammasome still remain elusive.

HMGB1, a highly conserved nuclear DNA-binding protein that stabilizes nucleosomes and regulates gene expression, will be sensed as an endogenous danger signal by the immune system when it occurs at the “wrong” place, and thus contributing to the development of inflammatory diseases (50, 51). Our previous findings supported HMGB1 as a crucial driver of macrophage inflammatory response (41). Substantial evidence also suggests that HMGB1 acts as a trigger of inflammasome activation, and high levels of HMGB1 have been observed in CHB patients (52, 53). In present investigation, our data showed that EGCG downregulated the serum HMGB1 level in rcccDNA mice, which was not due to the transcriptional inhibition of HMGB1 expression but the autophagic degradation of cytosolic HMGB1 in liver tissue. Autophagy is a lysosome-dependent degradation system that plays a fundamental role in cellular homeostasis (54), and has emerged as an important therapeutic target for various liver diseases. EGCG, a mimic of “starvation”, is a well-accepted autophagy inducer (44, 55). Further, our data demonstrated that blockage of autophagic flux reversed EGCG-mediated inhibition of HMGB1 release, NLRP3 inflammasome activation and the subsequent liver fibrosis progression in rcccDNA mice, supporting the autophagic degradation of HMGB1 as a key mechanism for EGCG’s therapeutic effect on HBV-induced liver fibrosis. These findings may strengthen the significance of HMGB1/NLRP3 inflammasome signaling in promoting liver injury and fibrosis during HBV infection. However, it is also reported that HMGB1 *alone* is a weak inducer of immune responses (51, 56), indicating that the activation of macrophage inflammasome by HMGB1 may require synergy with other pathological agents, which deserves further investigation.

Our further data showed that EGCG-mediated autophagic flux mainly happened in hepatocytes in rcccDNA mice. Furthermore, EGCG-induced autophagic flux in hepatocytes was found to correlate positively with the inhibition of liver injury and fibrosis, suggesting hepatocytes as the primary target of EGCG to exert its therapeutic effects. However, there are also studies suggesting EGCG as an autophagy inducer in LPS-treated macrophages, and thus leading to the degradation of HMGB1 (27, 29). The exact reason for this discrepancy remains unclear, which may be due to that HBV-infected hepatocytes rather than KCs are the primary cells for HMGB1 release as we demonstrated above, while LPS is known to preferably act on macrophages and stimulate macrophage inflammasome activation (57). EGCG has been reported to induce the aggregation of cytoplasmic HMGB1 protein by stably binding to HMGB1, which subsequently stimulates the autophagic degradation (29, 58). Therefore, in the presence of HBV infection, higher levels of cytoplasmic HMGB1 might lead to the increased autophagic flux in hepatocytes than in KCs when they are treated with EGCG. It is also possible that the inhibitory effect of EGCG on HMGB1 release was attributed to its inhibition of HBV replication. Nonetheless, our parallel experiments demonstrated that blockage of autophagic flux could significantly reverse the effect of EGCG on HMGB1 degradation while had little effect on its inhibition of HBV replication. Additionally, some injurious factors, besides HBV, may also contribute to the upregulation of cytoplasmic HMGB1 in hepatocytes during fibrosis progression. Collectively, we suggested autophagic degradation rather than HBV inhibition as the primary mechanism for EGCG-mediated suppression of HMGB1 release from hepatocytes. Of interest, our previous study has demonstrated that the activation of STING signaling stimulated autophagic flux in macrophages to suppress inflammasome activation, thereby leading to the alleviation of HBV-induced liver injury and fibrosis (15). We therefore proposed that a combined therapy with EGCG and STING agonist may synergistically ameliorate HBV-induced liver fibrosis *via* activating autophagy in both hepatocytes and macrophages, which deserves further investigation.

## 5 Conclusions

**Figure 7:**
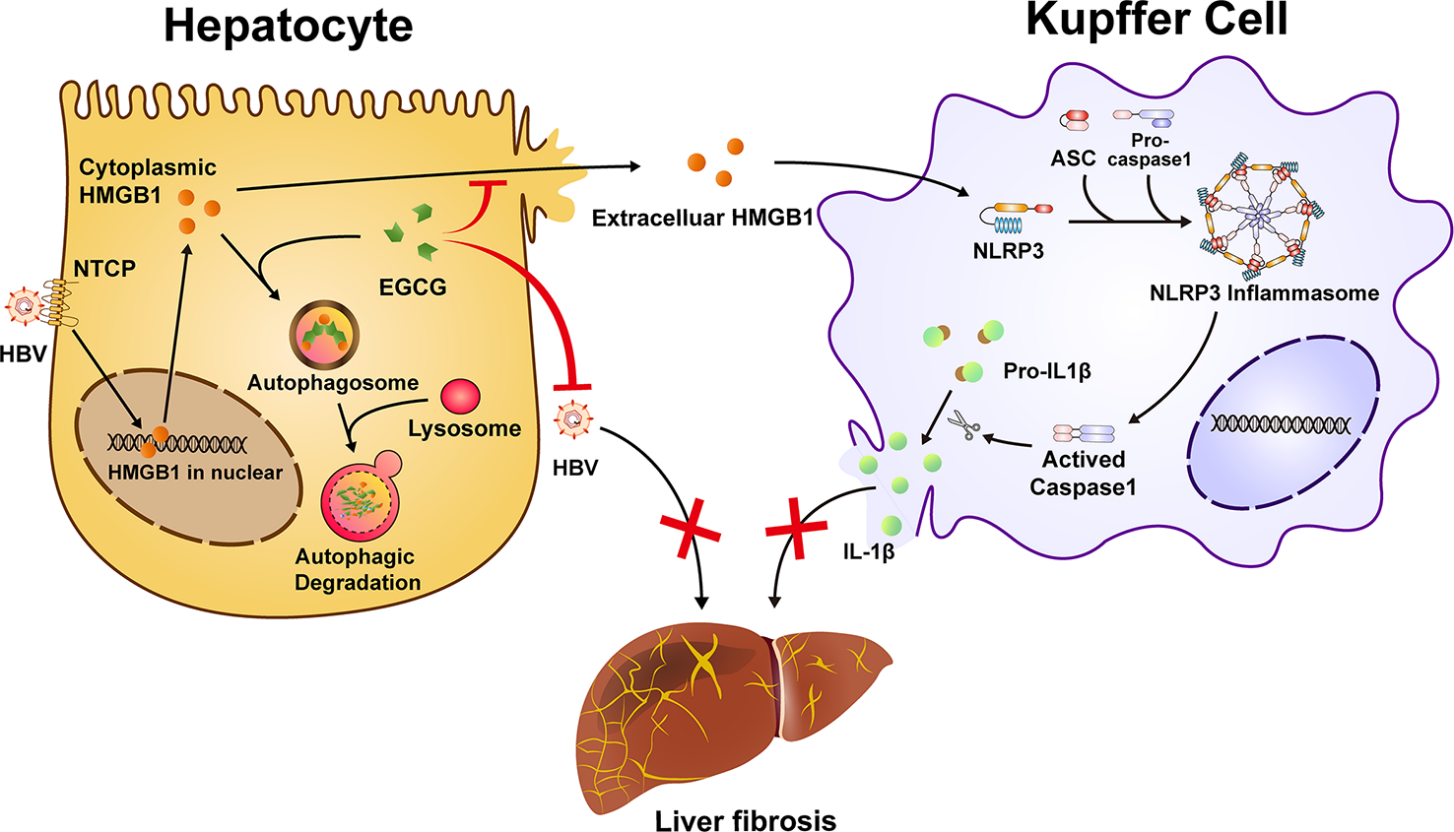
Schematic illustration of EGCG protects against HBV-induced liver injury and fibrosis *via* autophagic degradation of hepatic HMGB1. HBV triggers the nucleus-cytoplasmic translocation of HMGB1 and its following secretion from hepatocytes. Extracellular HMGB1 further participates in the activation of macrophage inflammasome, a critical aggravator of liver fibrosis. EGCG induces the autophagic degradation of cytoplasmic HMGB1 in hepatocytes and reduce its extracellular secretion, thus inhibiting NLRP3 inflammasome activation in Kupffer cells. Together, besides its inhibition of HBV replication, EGCG treatment ameliorates HBV-induced liver injury and fibrosis *via* inhibiting extracellular HMGB1-mediated macrophage inflammasome activation.

In summary, our study shows that EGCG could efficiently attenuate HBV-induced liver injury and fibrosis. Mechanistically, besides its inhibition of HBV, EGCG treatment reduces HMGB1 secretion from hepatocytes *via* inducing autophagic flux, which further inhibits macrophage NLRP3 inflammasome activation and the subsequent hepatic injury and fibrosis (**Figure 7**). These data demonstrate that EGCG administration may present as a promising preventive and therapeutic strategy for HBV-induced fibrosis.

## Data availability statement

The original contributions presented in the study are included in the article/**Supplementary Material**. Further inquiries can be directed to the corresponding authors.

## Ethics statement

The animal study was reviewed and approved by Animal Ethics Committee of Fudan University.

## Author contributions

BG, YL, XZ conceived and supervised the study. BG, MJH, and TC participated in the study design and analyzed the data. MJH, TC, ZW, YF, RS, MYH, SF and CC performed the experiments. BG, MJH, and TC wrote the manuscript. All authors contributed to the article and approved the submitted version.
