# Supplementary material for: Inhibition of macrophages inflammasome activation via autophagic degradation of HMGB1 by EGCG ameliorates HBV-induced liver injury and fibrosis
Source: Front Immunol. 2023 Apr 14;14:1147379. doi: 10.3389/fimmu.2023.1147379 (PMC10140519; doi:10.3389/fimmu.2023.1147379)
Supplement: Supplementary file 1 [file DataSheet_1.pdf]

Supplementary Figures and Tables

Supplementary Figure 1

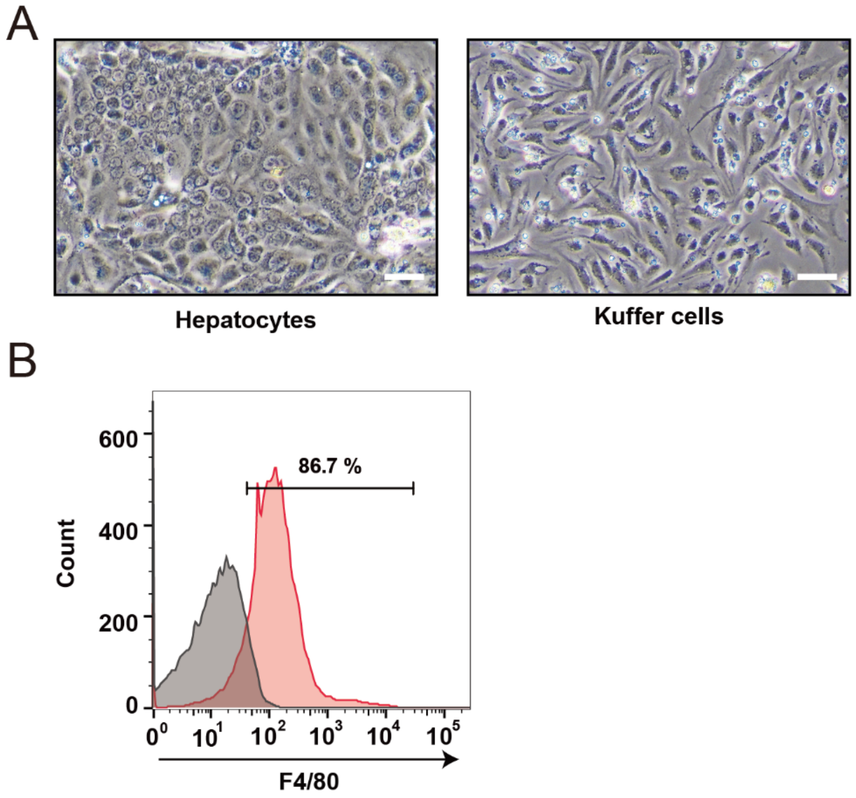

Supplementary Figure 1. Characterization of mouse KCs/Determination of morphology for isolated hepatocytes and Kupffer cells and purity for F4/80$^+$ Kupffer cells. (**A**) Hepatocytes and Kupffer cells were isolated from experimental mice and cultured *in vitro*. 3 days later, morphology of hepatocytes and Kupffer cells were determined by microscopy. Scale bar: 50 µm. (**B**) The isolated Kupffer cells were blocked and incubated with PE-Cy5.5-conjugated anti-F4/80 mAb, then the percentage of F4/80-positive cells was examined by flow cytometry.

Supplementary Figure 2

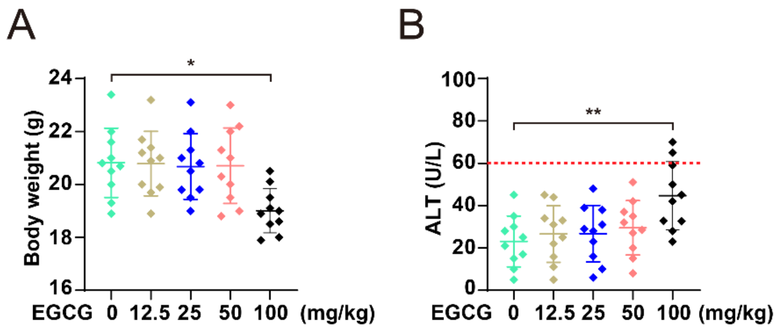

Supplementary Figure 2. The effect of different concentrations of EGCG on the body weight and ALT level in rcccDNA mice. Alb-Cre Tg mice were treated daily with i.p. injection of different doses of EGCG (12.5, 25, 50 and 100 mg/kg). 42 days later, the body weights **(A)** and the serum levels of ALT **(B)** were determined. Data are shown as mean ± SEM and compared by one-way analysis of variance (ANOVA). **P* < 0.05, ***P* < 0.01.

Supplementary Figure 3

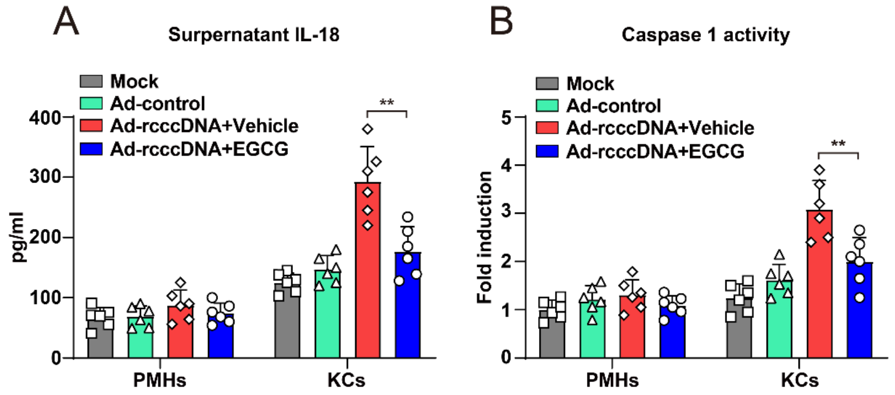

Supplementary Figure 3. EGCG suppresses IL-18 secretion and the lysate caspase-1 activity of KCs, rather than that of PMHs, in rcccDNA mice. PMHs and KCs were isolated from control and rcccDNA mice treated with or without EGCG (*n* = 6), and the supernatant IL-18 levels (**A**), and lysate caspase-1 activity (**B**) in PMHs and KCs were determined by ELISA and caspase-1 activity assay, respectively. Data are shown as means ± SEM and are compared by unpaired Student's t-test. ***P* < 0.01.

Supplementary Figure 4

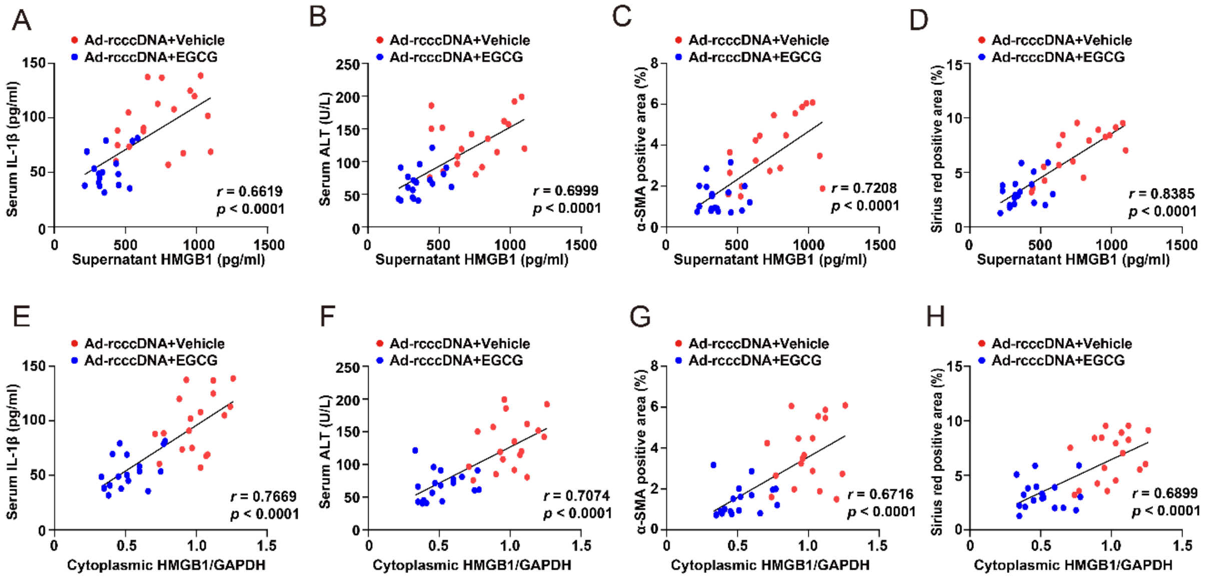

Supplementary Figure 4. The downregulation of supernatant and cytoplasmic HMGB1 in PMHs shows positive correlations with the EGCG-mediated attenuation of the severity of HBV-induced liver fibrosis. (**A** to **D**) Correlation analysis of supernatant HMGB1 levels in isolated PMHs with serum IL-1β (**A**), serum ALT (**B**), α-SMA positive area (**C**), and Sirius Red positive area (**D**), respectively in rcccDNA mice treated with or without EGCG. (**E** to **H**) Correlation analysis of relative cytoplasmic HMGB1 protein levels in isolated PMHs with serum IL-1β (**E**), serum ALT (**F**), hepatic α-SMA positive area (**G**), and hepatic Sirius Red positive area (**H**), respectively in rcccDNA mice treated with or without EGCG.

Supplementary Figure 5

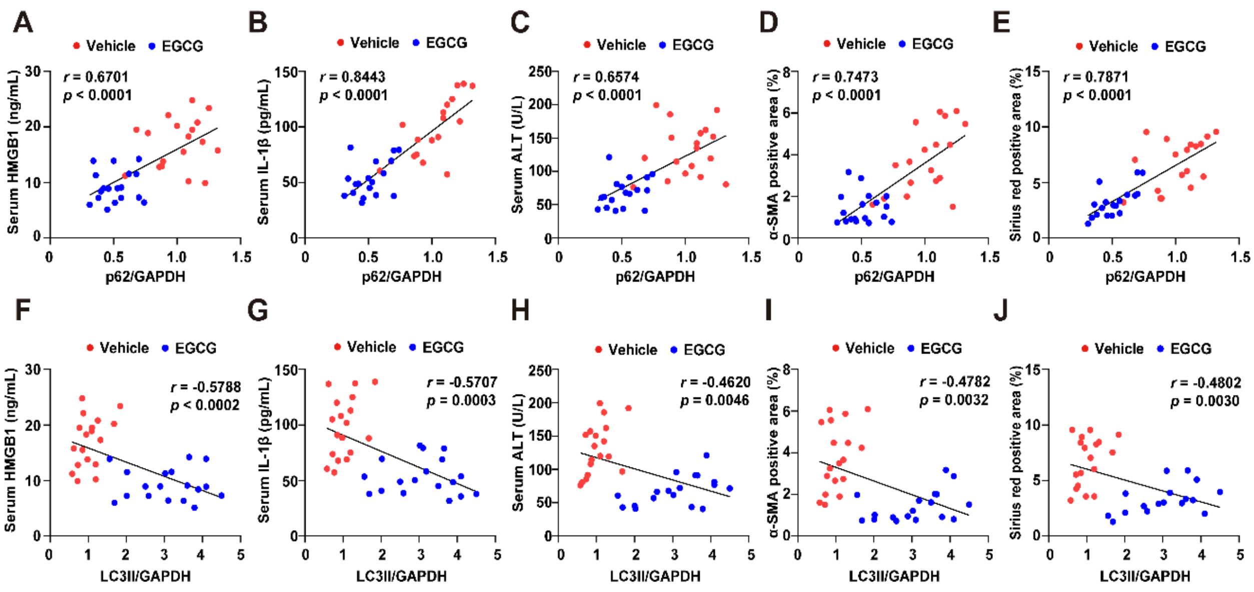

Supplementary Figure 5. **The extent of autophagic flux in PMHs is significantly correlated with the EGCG-mediated attenuation of the severity of HBV-induced liver fibrosis.** (**A** to **E**) Correlation analysis of relative p62 protein levels in isolated PMHs with serum HMGB1 (**A**), serum IL-1β (**B**), serum ALT (**C**), hepatic α-SMA positive area (**D**), and hepatic Sirius Red positive area (**E**), respectively in rcccDNA mice treated with or without EGCG. (**F** to **H**) Correlation analysis of relative LC3-II protein levels in isolated PMHs with serum HMGB1 (**F**), serum IL-1β (**G**), serum ALT (**H**), hepatic α-SMA positive area (**I**), and hepatic Sirius Red positive area (**J**) in rcccDNA mice treated with or without EGCG.

Supplementary Figure 6

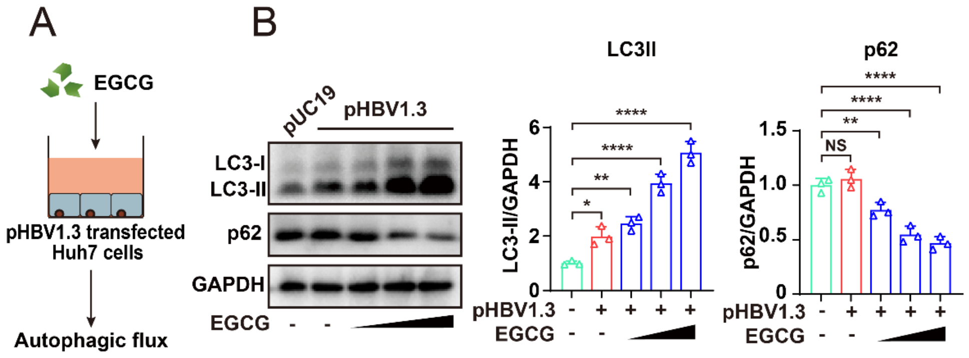

Supplementary Figure 6. EGCG induces autophagic flux in pHBV1.3-transfected Huh7 cells. (A) Huh7 cells were transfected with HBV replication-competent plasmids HBV1.3 (pHBV1.3) or control empty vector pUC19 for 48 hours, followed by the treatment of an increasing dose of EGCG (12.5 μM, 25 μM, 50 μM) for another 24 hours. **(B)** Protein levels of LC3-II and p62 were determined by Western blotting. Relative protein levels of LC3-II and p62 were determined by densitometric analysis, and the value from control group was set at 1.0. All data are shown as mean ± SEM and compared by one-way analysis of variance (ANOVA). $*P < 0.05$, $**P < 0.01$, $****P < 0.0001$. NS, no significance.

Supplementary Figure 7

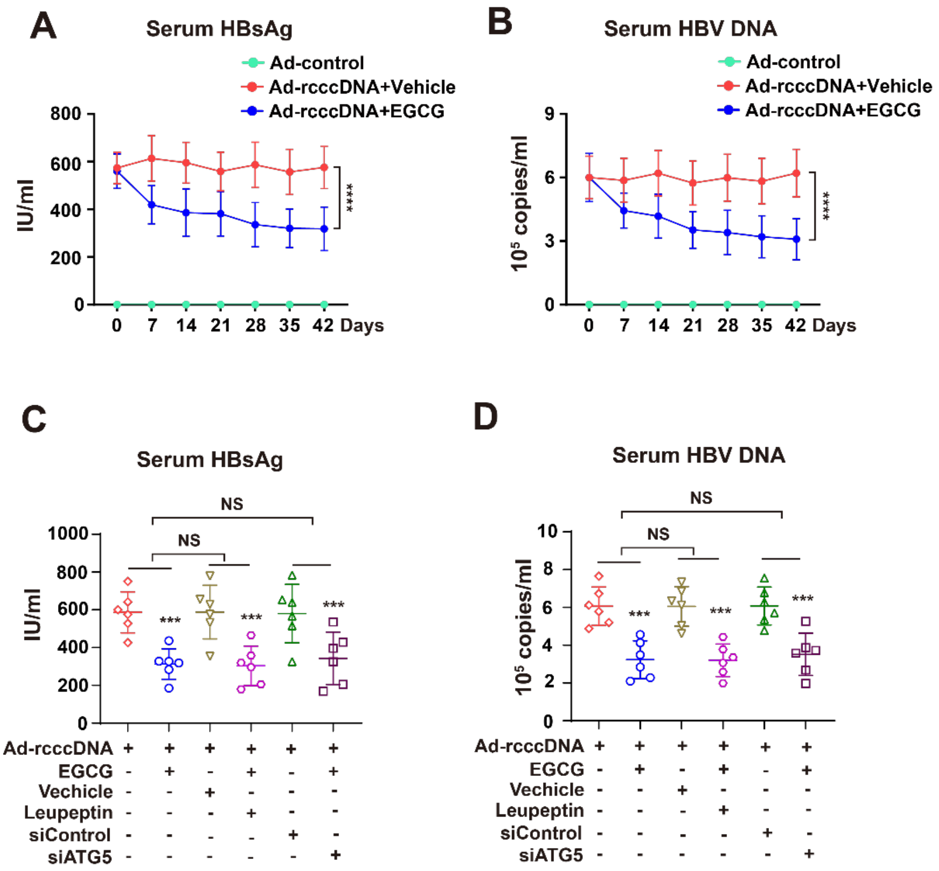

Supplementary Figure 7. Blockage of autophagic flux did not have significant effect on EGCG-mediated inhibition of HBV replication. **(A, B)** The rcccDNA mice were treated with EGCG (25 mg/kg) dail7y for 42 days (n = 6). Levels of serum HBsAg (**A**) and HBV DNA (**B**) were examined by ELISA and qPCR, respectively. **(C, D)** The rcccDNA mice were treated with EGCG (25 mg/kg) daily for 42 days through i.p. injection in the presence of leupeptin (20 mg/kg) or siATG5 siATG5 (2 mg/kg), followed by the determination of HBsAg **(C)** and HBV DNA **(D)** in mice sera (n = 6) as in A and B. The differences within and between groups were compared by Student's t-test and two-way analysis of variance (ANOVA), respectively. ***P < 0.001, ****P < 0.0001. NS, no significance.

Supplementary Table 1: Real-time PCR Primer Sequences

Gene	Forward sequences (5'-3')	Reverse sequences (5'-3')
α-SMA	GTCCCAGACATCAGGGAGTAA	TCGGATACTTCAGCGTCAGGA
Col 1a1	GAAACCCGAGGTATGCTTGA	GACCAGGAGGACCAGGAAGT
TIMP1	CTTGGTTCCCTGGCGTACTC	ACCTGATCCGTCCACAAACAG
MMP2	TTTGCTCGGGCCTTAAAAGTAT	CCATCAAACGGGTATCCATCTC
HMGB1	GGCGAGCATCCTGGCTTATC	GGCTGCTTGTCATCTGCTG
GAPDH	CTCTGGAAAGCTGTGGCGTGATG	ATGCCAGTGAGCTTCCCGTTCAG

Supplementary Table 2: Primary antibodies used in the present investigation

Antibody	Manufacturer
NLRP3	Abcam, ab263899
Caspase-1	Cell Signaling Technology, #3866
IL-1β	Bioworld Biotechnology, BS6067
GAPDH	Bioworld Biotechnology, AP0063
HMGB1	Bioworld Biotechnology, BS1918
Lamin B	Bioworld Biotechnology, AP6001
HBcAg	Abcam, ab8639
LC3	Sigma, #L7543
p62	Cell Signaling Technology, #88588
α-SMA	Cell Signaling Technology, #56856
ATG5	Cell Signaling Technology, #12994
