# Supplementary figures and images for: Inhibition of macrophages inflammasome activation via autophagic degradation of HMGB1 by EGCG ameliorates HBV-induced liver injury and fibrosis
Source: Front Immunol. 2023 Apr 14;14:1147379. doi: 10.3389/fimmu.2023.1147379 (PMC10140519; doi:10.3389/fimmu.2023.1147379)

**Supplemental information: (original Western blots)**

Fig. 2


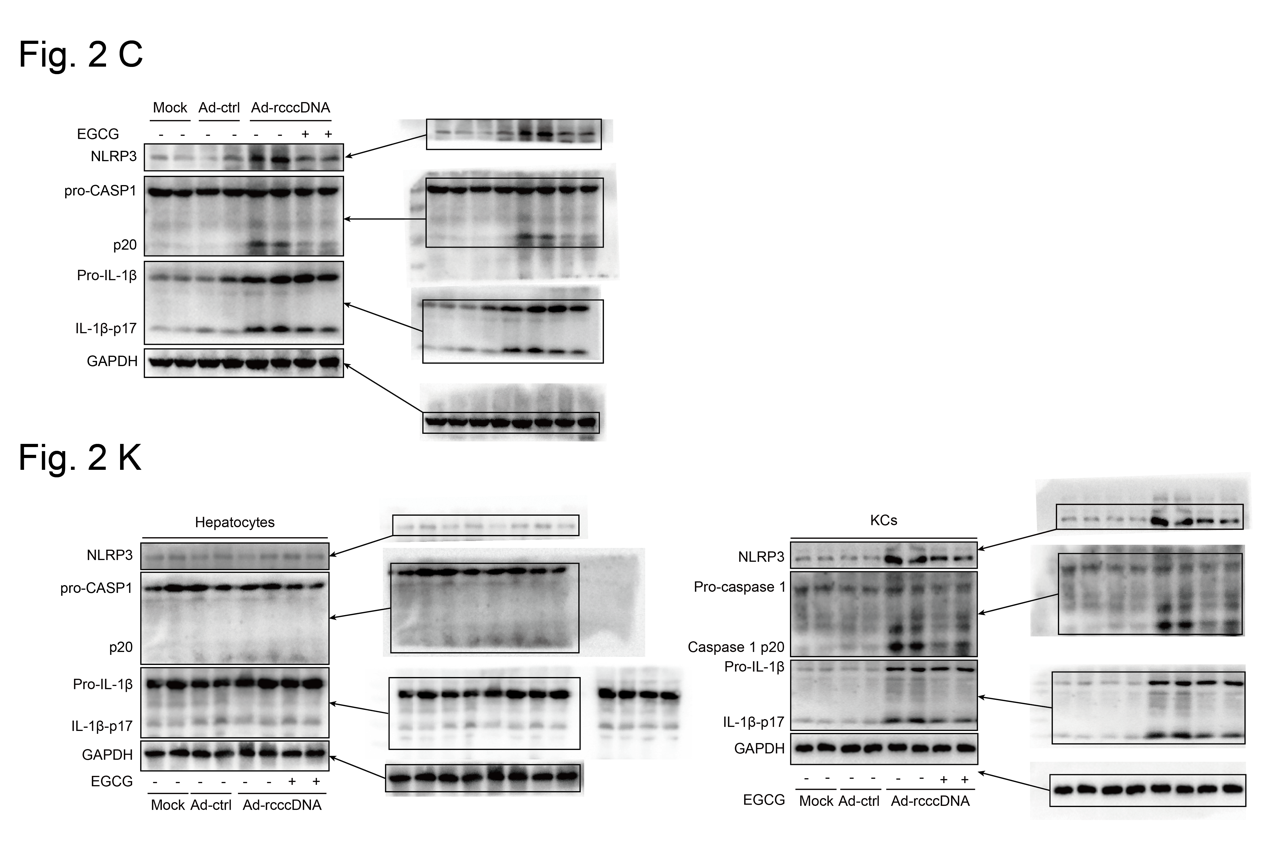


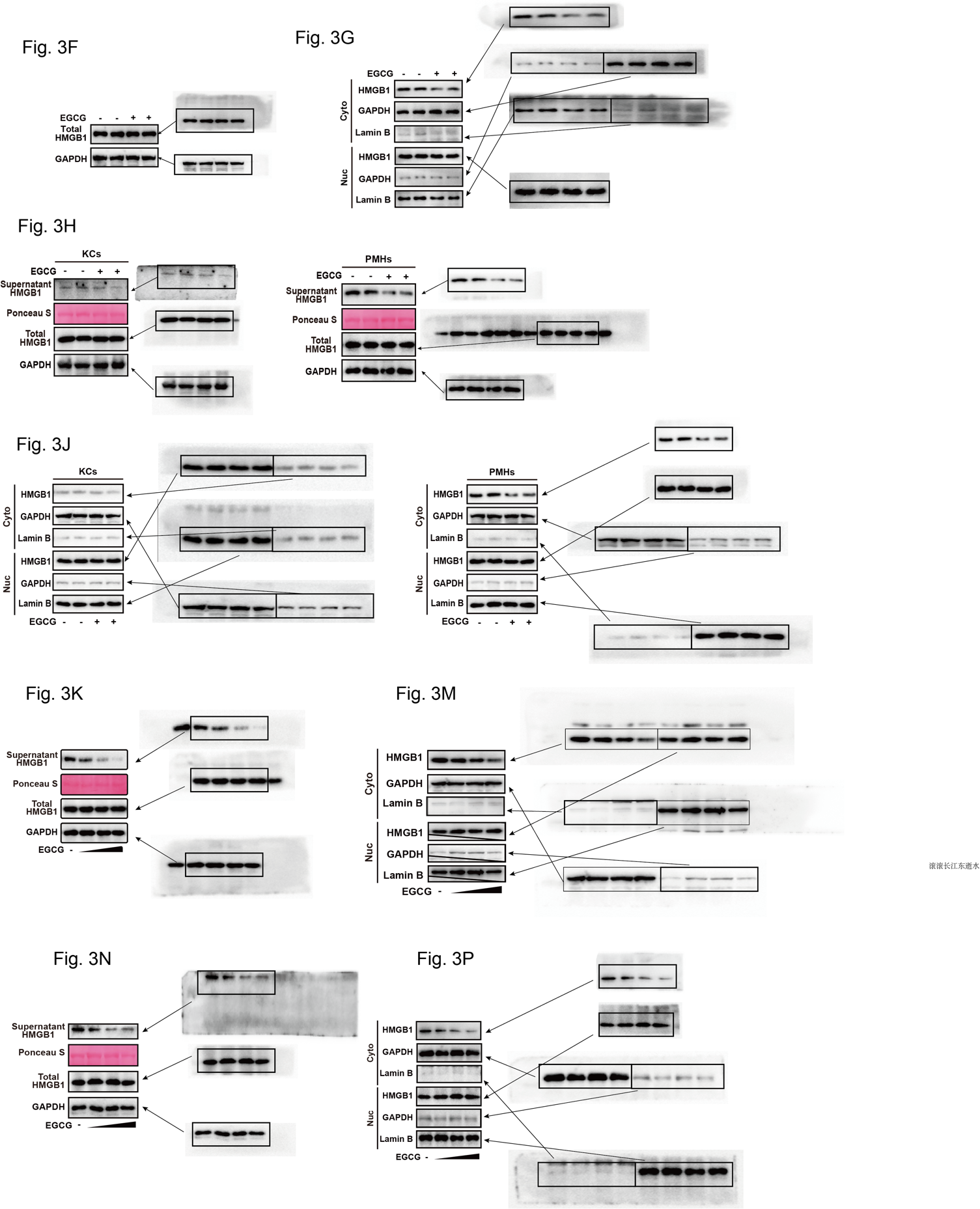
Fig. 3

Fig. 4


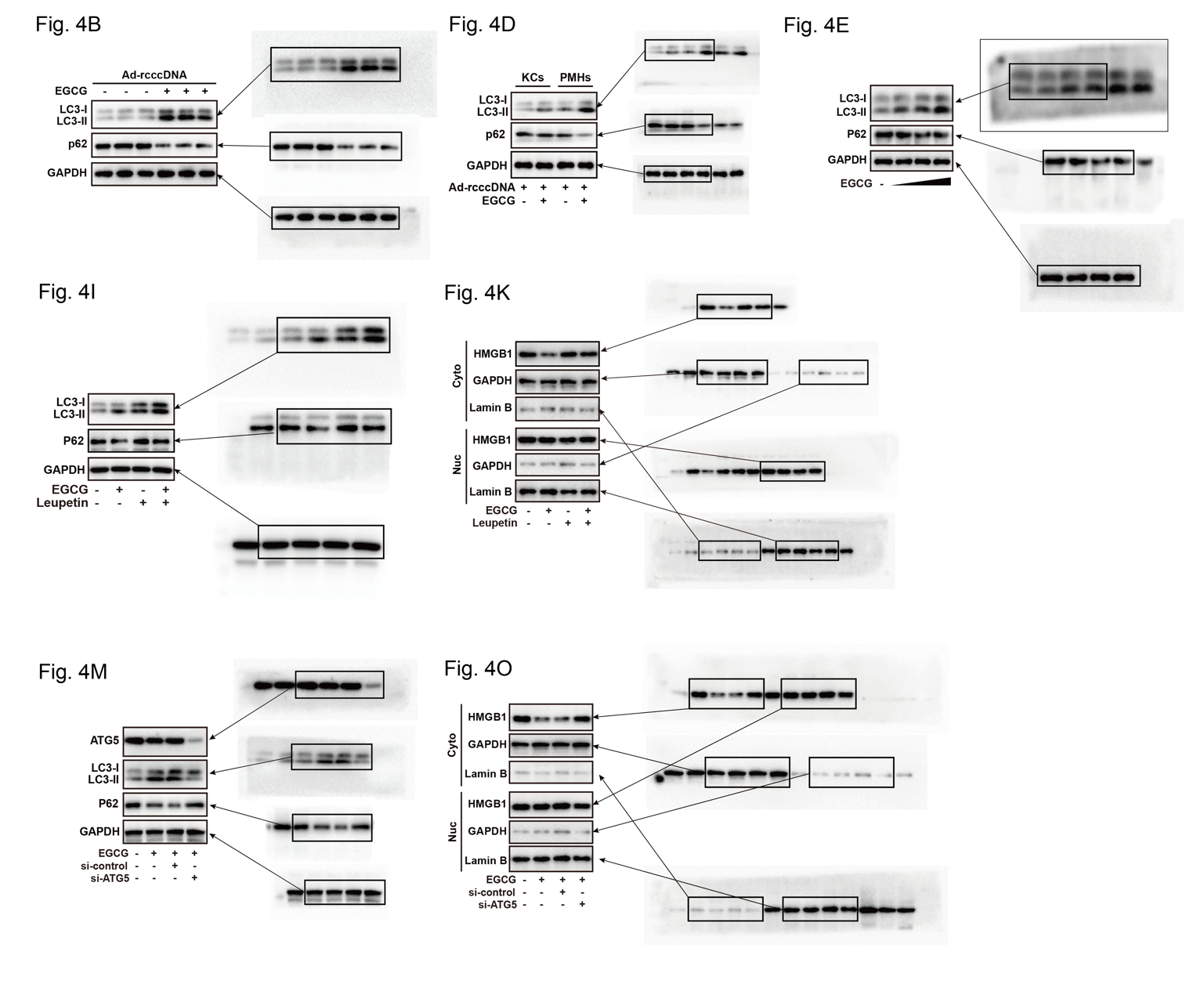


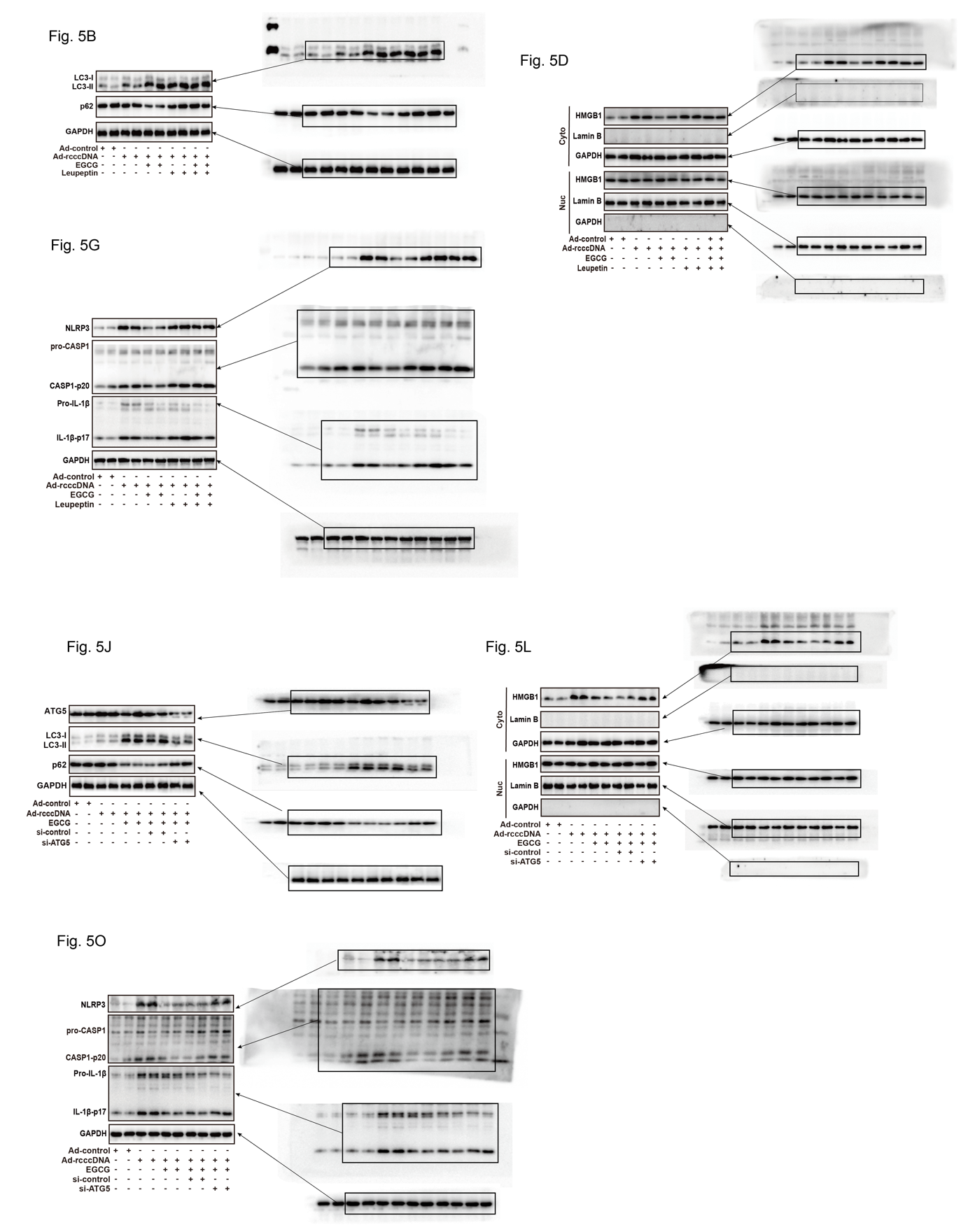
Fig. 5

Supplement: Supplementary file 2 [file DataSheet_2.docx]
